# Sertoli Cells Only Syndrome - Case Report

**DOI:** 10.5935/1518-0557.20200078

**Published:** 2021

**Authors:** Ana Bartmann

**Affiliations:** 1 Universidade de Ribeirão Preto - UNAERP - Ribeirão Preto - SP

**Keywords:** Exclusive Sertoli Cell Syndrome, azoospermia, IVF

## Abstract

Exclusive Sertoli Cell Syndrome (ESCS) is a rare condition that has male infertility as its main consequence. It is one of the most serious forms of non-obstructive azoospermia, with a poor reproductive prognosis. In some cases, however, such as the type II of the syndrome, sperm can be recovered through testicular puncture and subsequent ICSI, with a 13% success rate. This article aims to report the case of an azoospermic 35-year-old patient, with no other significant changes in complementary exams. After percutaneous puncture of the epididymis and biopsy with no sperm, we diagnosed ESCS, and indicated IVF with donor semen.

## INTRODUCTION

Exclusive Sertoli Cell Syndrome, also called germ cell aplasia or Del Castillo Syndrome, is a rare condition that results in male infertility (Gat *et al*., 2010; Behre *et al*., 2015). In general, the patient is male with normal external genitalia, well-developed secondary sexual characteristics and azoospermia (Hanmayyagari *et al*., 2015). It is one of the most serious forms of non-obstructive azoospermia, and may be associated with other clinical manifestations in some cases (Kavoussi *et al*., 2019; Paduch *et al*., 2019). However, the main complaint that makes the couple seek medical advice is the unsuccessful attempt at pregnancy (Kim *et al*., 2015).

It is characterized by the exclusive presence of Sertoli cells (without germ cells) in seminiferous tubules, making spermatogenesis impossible (Paulis *et al*., 2017). Leydig’s interstitial cells are present and produce testosterone normally. It can be primary, characterized by a reduction in growth factors GDNF, FGF8 and BMP4 - that at low levels do not induce replication and the stimulus for the differentiation of spermatogenic stem cells into spermatogonia or, secondary, as Klinefelter’s Syndrome, exposure to toxins or chemicals, viruses, radiotherapy, trauma and varicocele (Gat *et al*., 2019; Behre *et al*., 2015; Kavoussi *et al*., 2019; Paduch *et al*., 2019; Stouffs *et al*., 2016; Nistal *et al*., 1990).

Sertoli cells secrete anti-Mullerian hormone (AMH), which promotes the regression of Müller’s ducts as the male fetus develops (Behre *et al*., 2015; Kim *et al*., 2015; Anniballo *et al*., 2011). They also secrete inhibin and activin, which regulate FSH secretion by the hypothalamus (Kim *et al*., 2015). Activin increases the FSH levels needed for semen production, while inhibin helps maintain testicular homeostasis (Kim *et al*., 2015). In general, the complaint is infertility (Jain & Halder, 2012). The physical examination is not enlightening, and, in some cases, there may be testicular atrophy (10-20 mL in volume) or bilateral varicocele, without other important findings (Gat *et al*., 2010; Kavoussi *et al*., 2019; Matsumoto & Bremner, 2016). There are no signs of feminization, such as gynecomastia, and all male characteristics are preserved - Hanmayyagari *et al*., 2015.

In clinical investigation, there is a high FSH level and preserved testosterone levels (Behre *et al*., 2015; Hanmayyagari *et al*., 2015). AMH and inhibin B concentrations are decreased, and the luteinizing hormone (LH) may be normal or elevated (Hanmayyagari *et al*., 2015). In the semen, we find azoospermia (in syndrome’s type I), and we can rarely find spermatogenic spots (in syndrome’s type II) (Behre *et al*., 2015; Paulis *et al*., 2017; Abofoul-Azab *et al*., 2019). The karyotype is male, with no changes; however, microdeletions may occur on the Y chromosome (Stouffs *et al*., 2016; Jain & Halder, 2012). All of these findings and changes are suggestive of Exclusive Sertoli Cell Syndrome, but the final diagnosis is made with testicular biopsy revealing a complete absence of germ cells and seminiferous tubules covered by Sertoli cells only (Gat et al., 2010; Matsumoto & Bremner, 2016).

The reproductive prognosis of affected patients is quite poor, as there is no way to treat the condition itself (Hanmayyagari *et al*., 2015; Kavoussi *et al*., 2019; Paulis *et al*., 2017). In most cases, donor’s semen is used when the couple is interested in pregnancy (Hanmayyagari *et al*., 2015). Although sperm can be recovered in type II of the syndrome during testicular puncture (testicular sperm extraction TESE), current research suggests that only 13% of men were successful in ICSI procedures (Hanmayyagari *et al*., 2015; Paulis *et al*., 2017; Stouffs *et al*., 2016). In the case of obtaining an embryo by ICSI, pre-implantation genetic diagnosis is indicated to rule out possible chromosomal malformations and printing disorders related to the fertilization process, such as Angelman and Prader-Willi Syndromes (Fertilitypedia, 2020; Pan *et al*., 2018). Moreover, some studies report that such patients have an increased risk of testicular cancer. Thus, routine clinical assessments in men with SCOS are important (Kavoussi *et al*., 2019; Paduch *et al*., 2019).

## OBJECTIVE

To report the case of a patient with Sertoli Cell Only Syndrome.

## MATERIALS AND METHODS

We evaluated the medical records of the couple and a ran a bibliographic review in the Pubmed and LILACS databases, using the terms “Sertoli cell only syndrome”, “azoospermia”, “infertility”, “Testicular Disorders”. The review was carried out between September and December 2019.

## CASE REPORT

A.K.G., male, 35 years old, came with his wife to the Human Reproduction Center/Ana Bartmann Clinic of the Ribeirão Preto University (UNAERP) due to primary infertility. At the time of the consultation, they brought several spermograms, all of them with azoospermia.

Azoospermia was investigated: FSH, LH, TSH, total and free testosterone, karyotype and testicular ultrasound. With the exams, we diagnosed obstructive azoospermia and indicated percutaneous epididymis puncture (PESA) (Table 1). The patient accepted more invasive procedures if no viable sperm would be found during the procedure.

**Table 1:** Results of the tests performed

EXAM	RESULTS	REFERENCE
Total Testosterone	204.84ng/dl	65-753ng/dl
Calculated Testosterone	5.33ng/dl	3.03-14.80ng/dl
LH	9.4mUI/ml	1.50-9.30mUI/ml
FSH	14.88mUI/ml	1.40-18.10mUI/ml
TSH	1.12µUI/ml	0.48-5.60µUI/ml
Prolactin	8.61ng/ml	2.1-17.7ng/ml
G-band karyotype (100 cell count)	46XY, without changes	
Scrotum US	Testicular cyst on the right. Small volume hydrocele on the right. Absence of varicose dilatation of the pampiniform and peritesticular plexus	

On the same day, after the woman’s oocyte retrieval, the patient’s testicular puncture was performed. As no sperm was obtained after multiple attempts, we chose to perform testicular biopsy by microsurgery. The anatomopathological result of the testicular biopsy was testicles with germ-cell aplasia. Hypotrophic seminiferous tubules with foci of sclerosis represented 10% of the samples and there was complete absence of germ cells.

We indicated fertilization of oocytes the captured with donor semen.

## DISCUSSION

In this report, we discuss the case of a patient with Sertoli cell-only syndrome, a rare and irreversible cause of male infertility. We diagnosed infertility by analyzing the semen that revealed azoospermia. However, the syndrome’s etiology is only defined through testicular biopsy in which normal Sertoli cells can be found, but without germ cells.

The importance of early correction of other testicular pathologies, such as cysts, varicocele or hydrocele, is highlighted, so as not to impair male reproductive function and consequent secondary infertility.

Regarding treatment, those cases are difficult to manage, since there is no effective and available therapy for the syndrome. Current possibilities include ICSI, in cases where some sperm are found in the testicular puncture (type II syndrome), and semen donation as in the case described.

Unfortunately, in our case, the patient reported great difficulty in accepting donor semen. The oocytes were frozen and the couple is undergoing psychological counseling.
